# Therapeutic Drug Monitoring of Anti-Thymocyte Globulin in Allogeneic Stem Cell Transplantation: Proof of Concept

**DOI:** 10.3389/fphar.2022.828094

**Published:** 2022-03-18

**Authors:** J.I. Meesters-Ensing, R. Admiraal, L. Ebskamp, A. Lacna, J. J. Boelens, C. A. Lindemans, S. Nierkens

**Affiliations:** ^1^ Princess Máxima Center for Pediatric Oncology, Utrecht, Netherlands; ^2^ Department of Pediatrics, University Medical Center Utrecht, Utrecht, Netherlands; ^3^ Center for Translational Immunology, University Medical Center Utrecht, Utrecht, Netherlands; ^4^ Stem Cell Transplantation and Cellular Therapies, Memorial Sloan Kettering Cancer Center, New York, NY, United States

**Keywords:** anti-thymocyte globulin (ATG), TDM (therapeutic drug monitoring), stem cell transplant (SCT), antibody, pediatrics–children

## Abstract

Anti-thymocyte globulin (ATG), a polyclonal antibody, is used in allogeneic hematopoietic cell transplantation (HCT) to prevent graft-vs.-host-disease (GvHD) and graft failure (GF). Overexposure to ATG leads to poor early T-cell recovery, which is associated with viral infections and poor survival. Patients with severe inflammation are at high risk for GF and GvHD, and may have active infections warranting swift T-cell recovery. As ATG exposure may be critical in these patients, individualized dosing combined with therapeutic drug monitoring (TDM) may improve outcomes. We describe the individualized dosing approach, an optimal sampling scheme, the assay to measure the active fraction of ATG, and the workflow to perform TDM. Using a previously published population pharmacokinetic (PK) model, we determine the dose to reach optimal exposures associated with low GvHD and rejection, and at the same time promote T-cell recovery. Based on an optimal sampling scheme, peak and trough samples are taken during the first 3 days of once-daily dosing. The fraction of ATG able to bind to T-cells (active ATG) is analyzed using a bio-assay in which Jurkat cells are co-cultured with patient’s plasma and the binding is quantified using flow cytometry. TDM is performed based on these ATG concentrations on the third day of dosing; subsequent doses can be adjusted based on the expected area under the curve. We show that individualized ATG dosing with TDM is feasible. This approach is unique in the setting of antibody treatment and may result in better immune reconstitution post-HCT and subsequently better survival chances.

## Introduction

Allogeneic hematopoietic cell transplantation (HCT) is a potentially curative treatment option for a plethora of hematological and non-hematological diseases. While the vast majority of adult patients receive an HCT for malignant underlying conditions, approximately 50% of pediatric patients have a non-malignant disease as transplant indication. This group includes immune deficiencies, of which part lack (part of) cellular immunity (e.g., severe combined immunodeficiency), while other patients with immune deficiencies present with immune dysregulation and hyperinflammation (e.g., hemophagocytic lymphohistiocytosis, chronic granulomatous disease, combined variable immune deficiency).

The main limitations of HCT in these patient groups include graft failure (GF), infections, and graft-versus-host-disease (GvHD). Without rigorous recipient T-cell depletion, the T-cell compartment is prone to activation causing a high risk of GF. On the other hand, patients with active inflammation and activation of antigen-presenting cells are susceptible to development of GvHD. As such, an allogeneic HCT in these patients is a relatively high-risk procedure.

Anti-thymocyte globulin (ATG) was introduced to prevent GvHD and GF after allogeneic HCT (Storek et al., 2015). It mainly depletes T-lymphocytes of the recipient and graft, which are important mediator cells in GvHD and rejection (Mohty, 2007). However, in line with the mode of action, too rigorous T-cell depletion of the graft may result in delayed or absent T-cell reconstitution after HCT, leading to an increased risk for viral reactivations (Admiraal et al., 2017). ATG therefore has a central role in outcome after HCT, and appears to have a delicate balance between its efficacy and toxicity (Admiraal et al., 2015a; Admiraal et al., 2016).

ATG is a remarkable drug from a pharmacological perspective. The drug infused is a rabbit-derived polyclonal IgG with antibodies against a variety of epitopes found on human cells. Main manufacturing methods include inoculating rabbits with either whole human thymus tissue (Thymoglobulin, Sanofi, France) or Jurkat cells (human T-cell lymphoblasts; Grafalon [previously known as ATG-Fresenius], Neovii, Switzerland). Horse-derived ATG, enriched after inoculation with human leukocytes (Atgam, Pfizer, NY, United States), is not frequently used for the indication of allogeneic HCT. The different brands are not bio-equivalent due to their different production processes. The fraction of infused IgG varies between brands and is reported to be ∼9% for Thymoglobulin (Regan et al., 2001). Moreover, the specificity and capacity to bind to certain surface molecules on both lymphocytes and myeloid cells is different between the two brands (Bourdage and Hamlin, 1995; Oostenbrink et al., 2019). Since the PK, the PD and possibly also the assay to quantify exposure may be brand-specific, we only focus on Thymoglobulin in the current paper. Any mentioning of ATG should be therefore read as “Thymoglobulin”. Moreover, as the pharmacodynamics of the active fraction of ATG predict clinical outcome, we only focus on this active fraction.

Using a fixed dose of ATG, the exposures are highly variable and unpredictable (Admiraal et al., 2015b). However, the population pharmacokinetics (popPK) of ATG have been elucidated in recent years (Admiraal et al., 2015b). Body weight and absolute lymphocyte counts (ALC) before the first dose of ATG have been identified as drivers of its pharmacokinetics. The popPK model can be used to calculate expected ATG exposures given the patient characteristics. Moreover, with blood samples, the model can be used to perform therapeutic drug monitoring (TDM) to gain optimal control over the ATG exposure in an individual patient.

The exposure of ATG in the setting of an allogenic HCT can be divided in the exposure before and after infusion of the stem cell product. The exposure before graft infusion is suggested to be pivotal for the pharmacological effects (i.e., prevention of GvHD and GF), while the exposure to ATG after graft infusion is associated with T-cell reconstitution (Admiraal et al., 2015a; Admiraal et al., 2016). As such, in the setting of hyperinflammation with high risk for GvHD and GF, the exposure to ATG before graft infusion is of importance. On the other hand, the most optimal exposure to ATG after graft infusion still allowing immune reconstitution, is suggested to depend mainly on the stem cell source (Admiraal et al., 2015a). Using individualized dosing of ATG based on body weight and ALC, we aim to achieve maximal protection against GvHD and GF, while still promoting early T-cell recovery. However, for patients suffering from immune deficiencies and/or hyperinflammation the exposure prediction may be less accurate due to a potential large variation in dysregulated ALC in blood vs. inflamed tissue.

The aim of this paper is to describe the process of individualized dosing and TDM of ATG in the setting of immune deficiencies with hyperinflammation. We describe steps involved, including the individualized dosing, the optimal sampling scheme, the assay for the active fraction of ATG, the therapeutic drug monitoring and the workflow to perform the aforementioned steps.

## Need for Individualized Dosing and TDM

This protocol was designed for patients to reach optimal exposures to ATG both before HCT (associated with graft failure and GvHD) and after HCT (associated with T-cell recovery and survival) (Admiraal et al., 2015a; Admiraal et al., 2016). As a first step, we evaluated the performance of standard pediatric dosing of ATG (Thymoglobulin, Genzyme) in most protocols, being a cumulative dose of 10 mg/kg given in 4 consecutive days starting day-5. We simulated four representative patients with the indication of hyperinflammation: a body weight of 25 and 50 kg with an absolute lymphocyte counts before the first dose of 2 × 10 (Admiraal et al., 2015b)/L and 4 × 10 (Admiraal et al., 2015b)/L. We performed a total of 1,000 simulations per scenario, with full interindividual variability and residual error. The percentage of virtual patients reaching optimal exposure is shown in Table 1. The attainment of optimal exposure is relatively low, especially for the pre-HCT AUC <20% for all scenario’s), while over 50% of cord blood recipients in each scenario are over-exposed. As such, there was a clear indication to develop an individualized dosing regimen. As the dosages needed were higher than those used in most protocols, we also performed TDM, both to have more control over the exposures and to have more safety measures in place.

**TABLE 1:** Attainment of desired pre- and post-HCT Area Under the Curve.

Body weight	ALC	Pre-HCT AUC (all sources)	Post-HCT AUC (cordblood)	Post-HCT AUC (bone marrow/peripheral blood)
25	2	9.4	30.2	80.7
25	4	6.3	48.9	90.5
50	2	19.3	21.1	68
50	4	13	38.7	83.3

Percentage of simulated patients reaching desired pre- and post-HCT AUC, for the indication of hyperinflammation (pre-HCT AUC, 60–120 AU*day/mL; post-HCT AUC, cordblood <10 AU*day/mL; post-HCT AUC, bone marrow/peripheral blood <50 AU*day/mL) using a standard dosing regimen of ATG: 10 mg/kg over four consecutive days, starting day-5. ALC: Absolute lymphocyte count before first dose of ATG.

## Patients Eligible for TDM and Simulation for the Calculated Optimal Dose

The patients for whom this protocol is developed include those with immune deficiencies with hyperinflammation, who are at a high risk for graft failure and/or GvHD and are dependent on fast immune reconstitution to prevent infection. This includes but is not limited to patients with hemophagocytic lymphohistiocytosis (HLH), chronic granulomatous disease (CGD) and common variable immunodeficiency (CVID). The decision to include a patient in this protocol is at the treating physician’s discretion.

**FIGURE 1:**
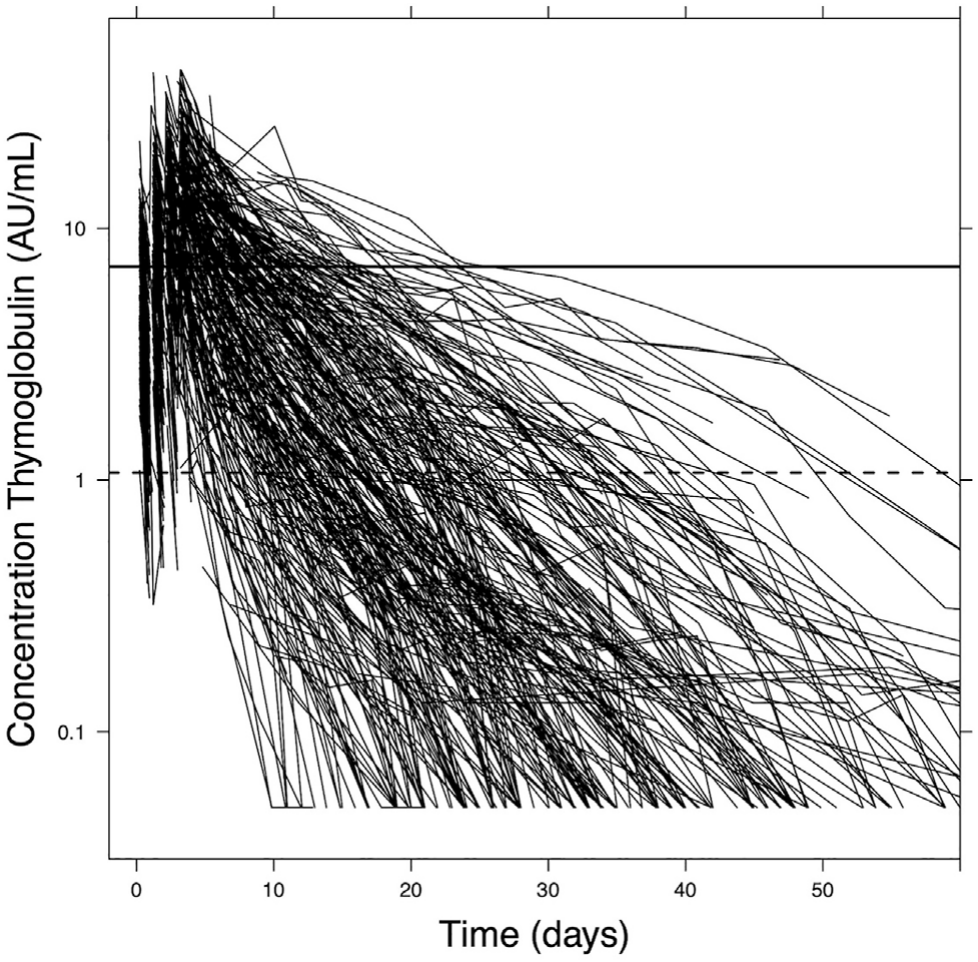
Active ATG plasma concentration data. The graph shows aATG concentrations over time in pediatric patients (*n* = 267) during the whole course of ATG treatment up to 60 days after first dosing. ATG clearance varies extensively between patients. Adapted from Admiraal et al., Lancet Haematology 2017.

Simulation studies are performed to find the optimal individualized dose based on the patient characteristics. We use the previously published popPK model for all exercises regarding the ATG dosing and TDM (Admiraal et al., 2015b). This model was developed in a pediatric population treated with ATG in the setting of allogeneic hematopoietic cell transplantation. Historically, the maximum dose of ATG given was 10 mg/kg, which led to a wide range of exposures due to the variable pharmacokinetics (Figure 1) (Admiraal et al., 2015a; Admiraal et al., 2015b). The popPK-model was made based on this population. Covariates included in the model were actual body weight and ALC before the first dose of ATG. The desired exposures were derived from previously published data (Admiraal et al., 2015a; Admiraal et al., 2016). We use NONMEM 7.5.0 (Icon, Hanover, MD, United States) with data visualization in R version 4.0.5 for all pharmacological analyses. For simulations, a total of 1,000 virtual patients were analyzed given the patient characteristics with full inter-individual variability to evaluate expected exposures to ATG.

In general, patients included in the protocol have a relatively high ALC, leading to a relatively high ATG clearance. Thus, the cumulative starting dose is usually higher than the accustomed 10 mg/kg. Patients with an ALC above 4 * 10^9^/L are capped at 4 * 10^9^/L, as this was the maximum ALC in the population the PK-model was built on. The optimal exposures were set at 60–120 AU*day/mL before graft infusion; after graft infusion the target was <10 AU*day/mL for cord blood recipients and <50 AU*day/mL for bone marrow grafts. These target exposures were derived from previous observations, where an ATG exposure before graft infusion >40 AU*day/mL was associated with lower GvHD and GF (Admiraal et al., 2015a). Under the assumption of increased (tissue) ALC in the hyperinflammatory patient, we determined the dose with the model to a desired exposure of ATG before graft infusion of 60–120 AU*day/mL. Exposures after HCT are set to those found in previous reports (Admiraal et al., 2015a; Admiraal et al., 2016). A cumulative dose of ATG is chosen so that median simulated exposures are well within the desired ranges. In order to maximize the safety of the procedure, the daily dose in the protocol was 5 mg/kg/day, this was capped to twice the daily dose in regular regimens, i.e., 2.5 mg/kg/day. Given the relatively high dose needed in most dosing scenarios (given the high ALC and thus high clearance), dosing of ATG is usually spread over six consecutive days. This also gives more time to perform the elaborate assay to measure active ATG concentrations and to adjust the dose for the last days. To ensure maximum exposure to ATG before graft infusion and minimize exposure after graft infusion, upfront ATG starting day-15 was chosen.

## Definition of the Optimal Sampling Scheme for TDM

The optimal sampling scheme was developed using stochastic simulations and estimations (SSE). The SSE was performed by comparing the selected dosing regimens to a full PK-profile with hourly simulated concentrations samples. In the first step, concentration-time profiles of a 1,000 patients were simulated with hourly sampling incorporating full inter-individual variability. Covariate values (body weight, baseline lymphocyte counts) were chosen from at random from the distribution that was observed in the patients based on whom the population PK-model was developed. Next, out of these simulated 1,000 patients, for each of the scenarios only the indicated times were selected. In the next step, we estimated all of the PK-parameters for each individual patient given their available samples in the scenario and their covariate values. In a last step, the root mean square error (RMSE) was calculated between the PK-parameter estimations of the full PK-profile and that of different dosing scenarios.

**FIGURE 2:**
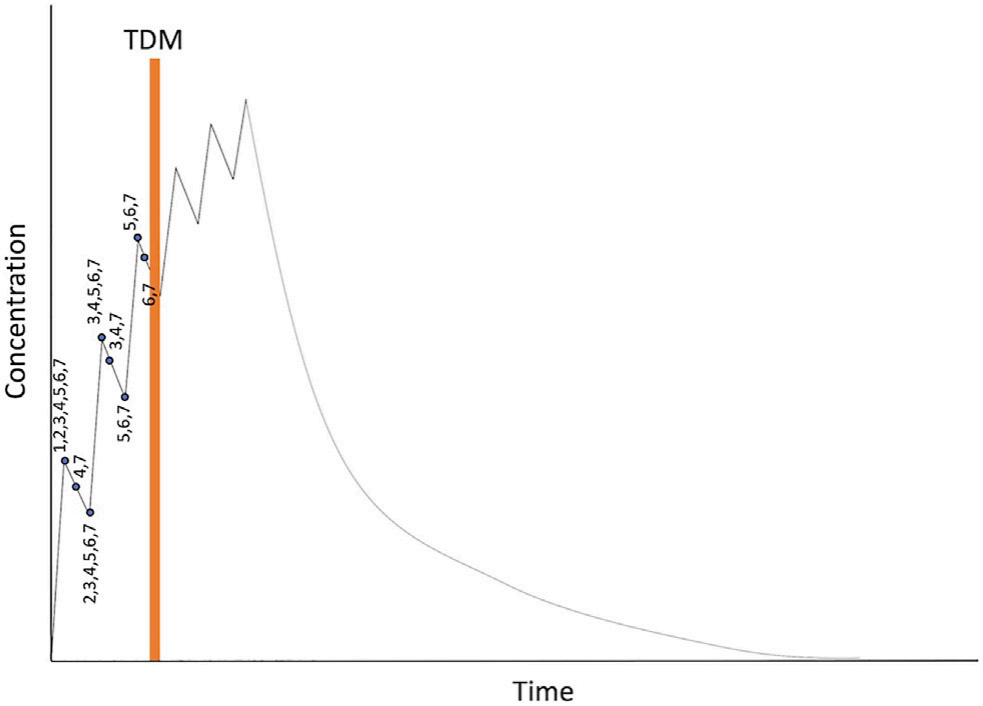
Overview of evaluated dosing regimens. Numbers represent the different dosing scenarios in Table 2.

Given that the maximum daily dose of ATG was set at 5 mg/kg from a safety perspective, patients received their total dose of ATG divided over up to six consecutive days. We evaluated dosing regimens where the assay for ATG will be performed during the 3rd or the 4th day of ATG, in both cases starting early in the morning. With this approach, the 4th (in case of day 3 TDM), 5th and 6th dose of ATG can be amended if necessary. This leaves the maximum window for potential sampling schemes from the start of the first dose up until the early morning of the day of assay (+68 h). We decided not to take any samples during infusion of ATG given the relatively slow distribution and clearance, combined with the potential inaccuracy in noting the times, and sampling a mixture of blood and infusion of drugs. The evaluated samplings schemes can be found in Table 2, with a graphical representation in Figure 2. RMSE’s showed that the scenario’s that take samples during 3 days of dosing with a sample on the early morning of the ATG assay (scenario 6 and 7) perform better in terms of clearance prediction compared to sampling during 1 or 2 days of dosing. Balancing the number of samples and amount of blood needed with the accuracy for all PK-parameters, the decision was made to sample according to scenario 6. As such, we draw blood at peak and trough during the first 3 ATG infusions, and one sample early in the morning of the 4th day of ATG for a total of six samples.

**TABLE 2:** RMSE of evaluated dosing regimens.

Scenario	Description	Clearance	Volume 1	Tm	Vmax	Km
Scenario 0	Hourly samples	0.28	0.26	7.55	1.73	17.88
Scenario 1	1 sample 1 day	2.43	1.23	16.61	1.80	17.88
Scenario 2	2 samples 1 day	2.25	1.20	13.53	1.79	17.89
Scenario 3	4 samples 2 days	1.34	0.92	13.29	1.79	17.89
Scenario 4	5 samples 2 days	1.04	0.92	11.93	1.79	17.89
Scenario 5	5 samples 3 days	1.36	0.80	13.29	1.79	17.89
Scenario 6	6 samples 3 days	0.59	0.82	12.72	1.79	17.89
Scenario 7	8 samples 3 days	0.58	0.81	11.32	1.79	17.89

Root mean square error (RMSE) after stochastic simulation and estimation for optimal sampling. A lower RMSE (range 0–∞) means less error in estimation of the parameter at hand. No interindividual variability is included on K21 and Tmax, therefore the RMSE, is 0 in all scenarios.

We tried to translate the inaccuracy of the different sampling schemes to the percentage of desired target attainment. The differences between actual and estimated AUC can be divided into two factors: a discrepancy between actual and estimated PK parameters, and the within-subject variability that may change the PK from dose to dose.

There is no contribution of within-subject variability in a potential inaccurate estimation, as this was explored but not identified in the modelling exercise. When trying to estimate the imprecision of the different sampling schemes, we tried developing a script that could automate the adjustment of the dose based on the TDM. Due to the non-linear elimination found in ATG, and the two separate desired optimal exposures (before and after HCT), we could not calculate the dose by multiplying AUC with clearance. We therefore are unable to present the percentage of desired target attainment in the presented sampling schemes.

## Therapeutic Drug Monitoring

During the days the patient is treated with ATG, the nurses register the exact times of starting and ending the ATG infusion, and the exact times on which the blood is drawn. At the set times, the samples are drawn from the central line and are sent to the lab where these are and frozen at −20 or −80°C until further use. The form with dosing and sampling times is sent to the clinical pharmacologists/pharmacist after completion. After performing the assay as described in the following paragraphs, the measured concentrations along with the patient characteristics, dosing details and actual dosing/sampling times are entered into a command-line based script. Based on these dosing- and sampling records and the patient characteristics, a NONMEM-ready database is compiled holding only the information for the individual patient using R version 4.0.5. Next, the data is run in NONMEM 7.5.0 ((Icon Devel-opment Solutions LLC, Hanover, MD, United States) using a previously developed population PK model (Admiraal et al., 2015b). Here, the population means are fixed to those found in the reported analysis with interindividual variability and residual error as was found in the report. Empirical Bayes estimates were calculated using the POSTHOC option with MAXEVAL = 0. AUC was calculated as a virtual third compartment in the PK-model.

The exposure to ATG before and after graft infusion is outputted by NONMEM, and along with a full concentration-time curve visualized in a time graph based on the measured and calculated concentrations and individual PK. The same script allows for adjustments to the dose and the number of dosages, as well as number of days between first dose of ATG and the time of graft infusion. Based on the individual pharmacokinetic parameters and the measured concentrations, the AUC before and after graft infusion is calculated when implementing dose adjustments. The target AUC did not differ between individualized dosing and TDM: 60–120 AU*day/mL before graft infusion and after graft infusion <10 AU*day/mL for cord blood recipients and <50 AU*day/mL for bone marrow grafts (Admiraal et al., 2015a; Admiraal et al., 2016). In case of ±25% adjustment in cumulative dosing, another session of TDM is advised on the 5th day.

## Development and Performance of a Quantitative Method for Measuring Active ATG

### Assay Protocol

**FIGURE 3:**
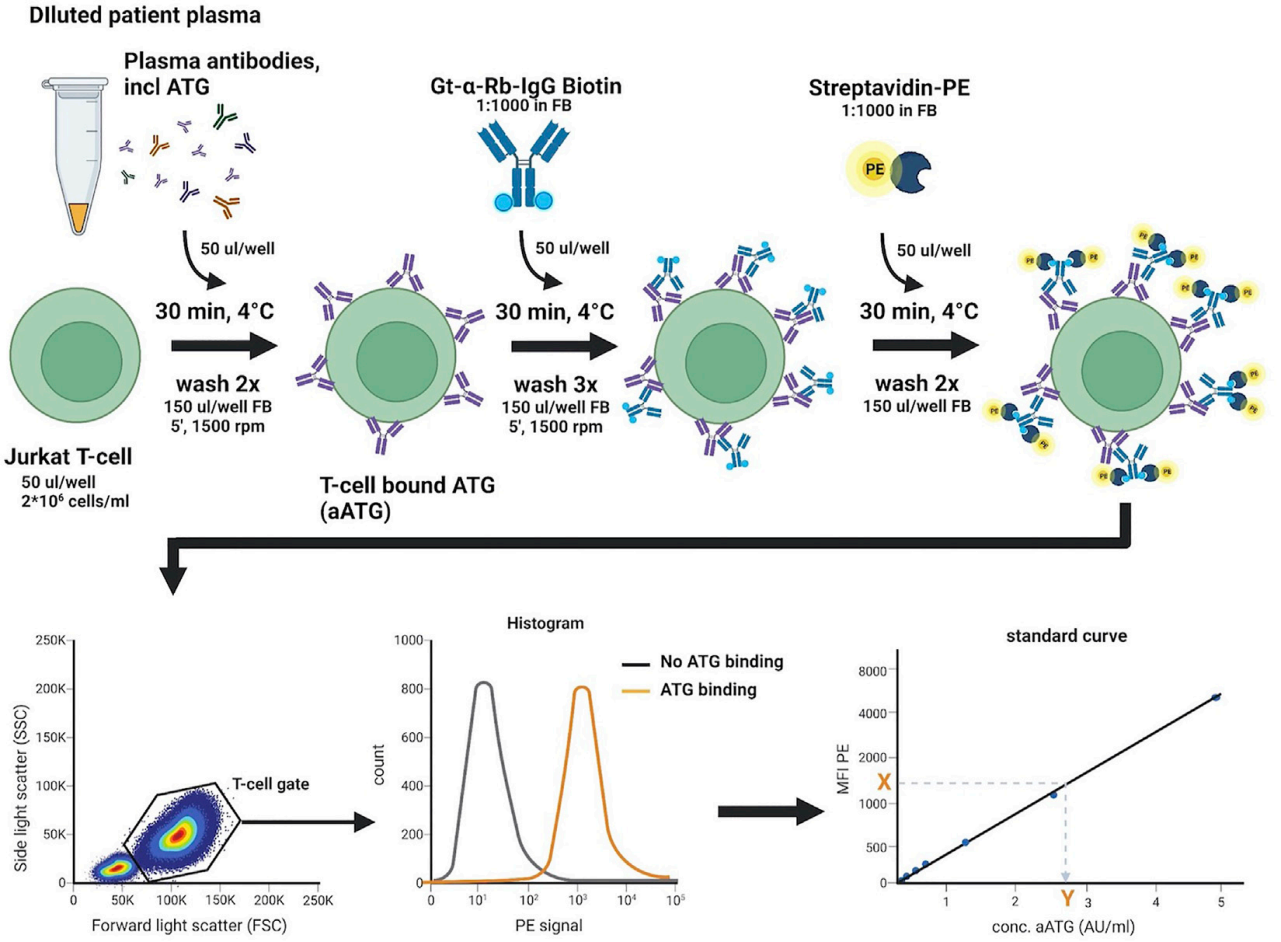
Active ATG binding assay. Standard curve, QC, negative control, and 4- and 8-fold diluted patient plasma samples are incubated in a 96-well U-bottom plate with target Jurkat T-cells, to allow for binding of aATG. Detection of bound aATG is done by subsequent incubation with a Gt-α-Rb-Ig-biotin antibody and streptavidin-PE. Cells are then analyzed on a FACS Canto II flow cytometer by acquiring 70 µl sample at a flow rate of 3 μl/s and intermittent mixing, measuring 10.000 events within the live T-cell gate. The median fluorescence intensity (MFI) of PE measured is then used to extrapolate the concentration of aATG (in AU/ml) in the patient sample from the standard curve. FB, FACS buffer (PBS/1%HSA). This figure was created with Biorender.com.

The assay to measure active ATG is based on an assay previously described by Rebello *et al.* (Rebello and Hale, 2002), where the concentration of an active compound in serum was quantified by measuring binding of this compound to a specific T-cell line using flow cytometry. Figure 3 shows a detailed description of the assay we developed.

We selected the Jurkat T-cell line as target for active ATG. Jurkat cells are maintained in RPMI 1640 medium (Fisher scientific, #11594506), supplemented with 10% (v/v) human serum albumin (HSA, Sanquin, #15522598), 1% (v/v) l-Glutamine (200 mM, Fisher scientific, #11500626), and 1% (v/v) penicillin/streptomycin (100x, Fisher scientific, #15140122) and cultured at 37°C and 5% CO_2_. Cells are expanded for a maximum of 10 passages, passaging cells every 2-3 days. On the day of measurement, the cells are harvested and diluted in PBS.

Binding of active ATG is detected by incubating 1*10^5^ cells with thawed patient samples that are filtered (0.45 µM filter plate, Millipore, #MSHVN4510) and diluted 4- and 8-fold in PBS to ensure active ATG levels detection within a quantification range for all samples. After incubation with samples, the cells are subsequently incubated with a Goat-anti-Rabbit IgG-Biotin antibody (Bio-connect, #111-065-045), followed by streptavidin-PE (BD, #554061) substrate to measure fluorescence on a FACS Canto II flow cytometer.

Active ATG concentrations present in patient samples are extrapolated from a standard curve using Thymoglobulin^®^ (Genzyme, #726816). A stock solution of 5000 AU/ml is prepared by dissolving the lyophilized product in PBS to a concentration of 5 mg/ml, which is aliquoted and stored at −80°C and diluted in PBS to create standards ranging from 5—0.005 AU/ml on the day of measurement.

### Assay Validation

Assay accuracy and precision: intra- and inter-assay variability, including intra- and inter-plate variability were determined using spiked QC samples that were created by preparing ATG stock solutions in PBS, which were then mixed 1:1 with ATG-naïve human plasma. ATG-naive human plasma also served as a negative control. An acceptable coefficient of variation (CV) for the bio-assay was defined to be below 25% or 30% for intra- and inter assay variability, respectively. Average CV values of the intra-assay variability was 10.7% for intra-plate and 9.3% for inter-plate samples, measured in at least 2 independent experiments. For the inter-plate variability this was 21.1%, measured over six independent experiments.

**FIGURE 4:**
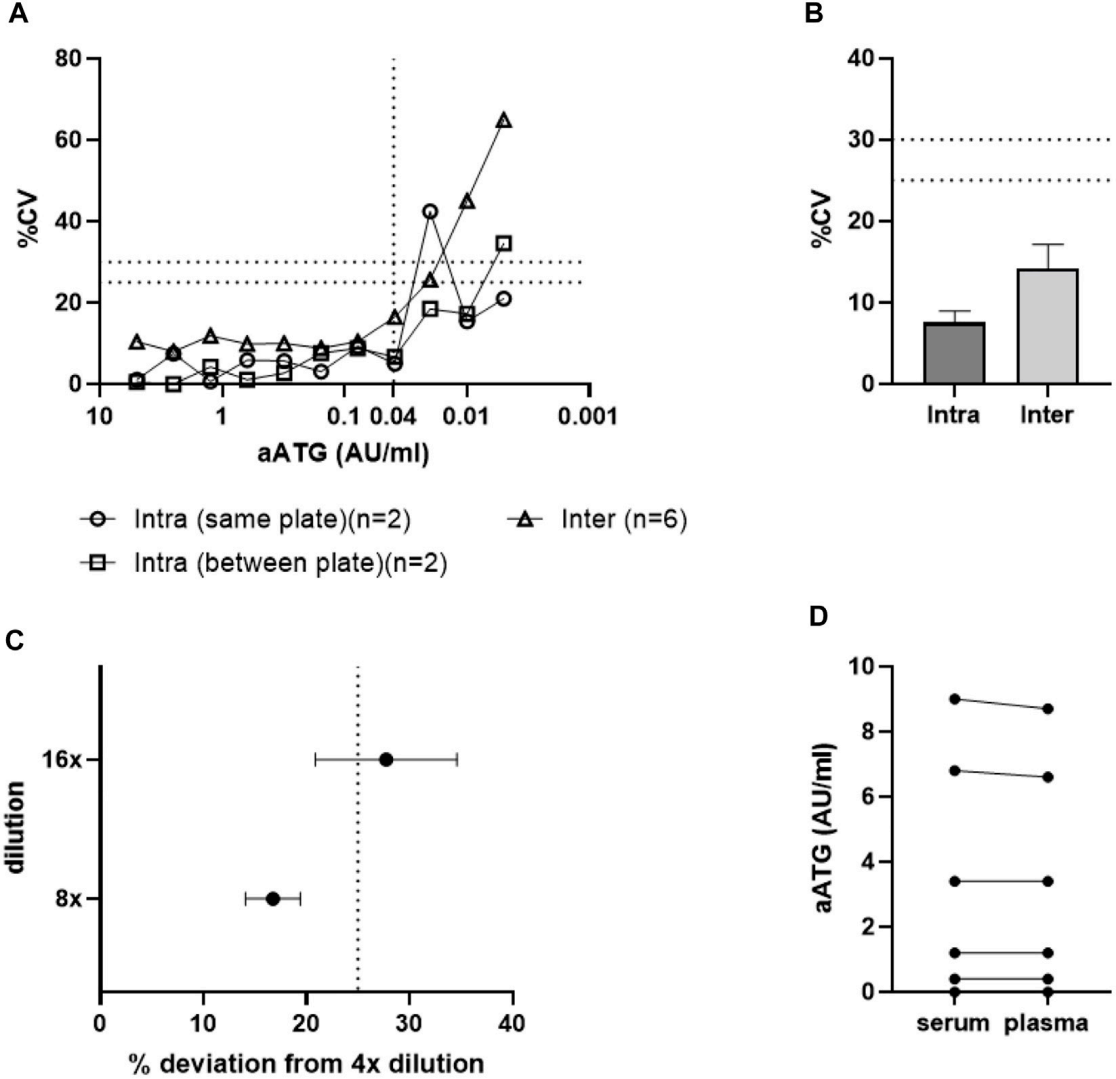
Active ATG binding assay validation. Graph A and B show results for assay accuracy and precision expressed in %CV. Results are shown for Intra- and inter-assay variability results of **(A)** standard curve samples and **(B)** patient samples (data shown as mean +SEM). The LLoQ was set at 0.04 AU/ml **(A)**. Graph **(C)** demonstrates that high patient sample dilutions (16-fold) show increased deviation above the threshold of 25% and should therefore be excluded from analysis (data shown as mean ± SEM). Dashed lines in graphs **(A–C)** represent maximal acceptable assay/sample variability expressed in %CV. Graph **(D)** demonstrates that both plasma and serum samples from the same patient show similar results in the aATG binding assay and can therefore both be used for analysis.

Based on individual CV values per standard concentration, the lower limit of quantification (LLoQ) was determined at 0.04 AU/ml (Figure 4A), as multiple samples with active ATG concentrations below 0.04 AU/ml exceeded the 25% CV limit. In addition to spiked standards, assay variability was also assessed using patient samples. For intra-assay variability the average CV was 7.6% with only 1 out of 29 samples not reaching the criterium of 25%. For the inter-assay this variability was 14.2%, with 2 out of 14 samples not reaching the criterium of 30% (Figure 4B).

As a final assessment of assay accuracy and precision, the influence of patient sample dilutions on assay outcome was investigated as patient samples taken both at peak and trough time points should be within the range of quantification. Patient plasma samples were diluted 4-, 8-, and 16-fold in PBS and deviations in concentration measured in the 8- and 16-fold dilutions compared to the 4-fold dilution were acceptable up to a bias of 25% (Figure 4C). We noticed that dilution did not show an exact linear relation with calculated ATG levels and that the 16-fold dilutions showed increased sample variation and should therefore not be included in the assay.

Overall, these results indicate that the assay is very robust with sample dilutions up to 8x and active ATG concentrations within the range of the standard curve and above the LLoQ.

### Analytical Specificity and Selectivity

To demonstrate assay specificity for the identification of active ATG in plasma, the assay was performed using spiked plasma samples of ATG-naive controls. Recovery of active ATG in these spiked plasma samples was 100%, compared to 0% in non-spiked plasma controls (data not shown). Sample variation was high in the 1.5 AU/ml samples, however overall, these data demonstrate that the assay can correctly quantify active ATG in plasma and that there was no non-specific detection of active ATG in control samples. It is of note that in patients receiving specific medication the measurements might potentially be interfered, but we could not find a compound-specific effect.

In addition to analytical specificity, selectivity of the assay was assessed by measuring active ATG levels in both plasma and serum samples of patients, taken at the same timepoint during ATG treatment. Figure 4E demonstrates that these different matrices do not influence assay outcome and that both patient materials can be used in this assay.

### Ethical Considerations

We used patient samples for the assay accuracy, which were collected after giving informed consent. We also used sera from healthy controls that were taken from volunteers. Institutional ethical committee approval for sample collection was given through trial numbers 11/063-k (patients) and 07/125 (healthy donors).

## Conclusion

This paper gives an overview of the pharmacology and the bio-assay needed to perform TDM on the polyclonal antibody ATG. We describe the processes of individualized dosing, the optimal sampling scheme, the actual TDM of active ATG, and present the optimized assay for measuring the active fraction of ATG. This protocol can be applied for difficult-to-treat patients, such as those with immunodeficiencies with hyperinflammation who are at a high risk for GF and GvHD, to check whether the prediction of exposure related to the given dose was accurate, and, if needed, to adjust the dose for the last administration(s). While this protocol may improve the control over the exposure to ATG, and thereby potentially clinical outcome, the assay and the need for pharmacokinetic analysis represent a limit in its application.

The main hurdles for setting up a protocol for TDM of active ATG include the complexity of the assay and the experience on using popPK for TDM. This elaborate description of the validated assay for quantifying ATG, using readily available reagents and cell lines, will support centers to implement this locally or a central lab should be defined that is able to perform the assay on the day of the day 3 sample (day 3 of ATG administration). As the described assay requires biological materials, results will be subject to a certain margin of error. To minimize assay variability, standardized batches of critical materials should be used (e.g., cells, buffers, ATG, and QC stock solutions) when implementing the assay for clinical use.

Another limitation may be the software and expertise on popPK that are used in the current protocol. In order to reduce the complexity of database building and running TDM using NONMEM, the popPK model was recently implemented in the software package InsightRx (InsightRx, CA, United States). This will make the simulation and modelling easier and more accessible.

The need for serotherapy in patients with hyperinflammation in the context of HCT for immune deficiencies is quite clear. Currently, rabbit ATG (Thymoglobulin, Grafalon) and alemtuzumab, an anti-CD52 monoclonal antibody, are available for this indication (Fox et al., 2018; Slatter et al., 2018; Chandra et al., 2021). Alemtuzumab is more potent, as reflected in a lower lympholytic level (Lane et al., 2014; Marsh et al., 2016), with a longer half-life compared to ATG (Admiraal et al., 2019). Therefore, when given in normal dosages at the same day relative to graft infusion, alemtuzumab may yield in more potent suppression of GvHD and GF, but potentially also leads to poor T-cell recovery. Still, alemtuzumab is currently used for the indication of hyperinflammation, with subcutaneous low dosing implemented to overcome slow or absent T-cell recovery (Bhatt et al., 2020). TDM of alemtuzumab is mainly focused on preventing underdosing (Arnold et al., 2021). From a pharmacokinetic perspective, exposure of ATG is easier to control given the higher clearance compared to alemtuzumab. A formal study comparing ATG and alemtuzumab, both with therapeutic drug monitoring, with endpoint PK-target attainment or clinical outcomes, would be needed to demonstrate which approach is superior.

In conclusion, we describe the assay to accurately measure active ATG concentrations, and the framework for TDM of ATG in an allogeneic HCT setting. This approach is unique in the setting of antibody treatment and may result in less complications, better immune reconstitution post-HCT and subsequently better survival chances.

## Data Availability

The original contributions presented in the study are included in the article/Supplementary Material, further inquiries can be directed to the corresponding author.
