# Corrigendum: Therapeutic drug monitoring of anti-thymocyte globulin in allogeneic stem cell transplantation: proof of concept

**DOI:** 10.3389/fphar.2024.1444228

**Published:** 2024-08-06

**Authors:** J. I. Meesters-Ensing, R. Admiraal, L. Ebskamp, A. Lacna, J. J. Boelens, C. A. Lindemans, S. Nierkens

**Affiliations:** ^1^ Princess Máxima Center for Pediatric Oncology, Utrecht, Netherlands; ^2^ Department of Pediatrics, University Medical Center Utrecht, Utrecht, Netherlands; ^3^ Center for Translational Immunology, University Medical Center Utrecht, Utrecht, Netherlands; ^4^ Stem Cell Transplantation and Cellular Therapies, Memorial Sloan Kettering Cancer Center, New York, NY, United States

**Keywords:** anti-thymocyte globulin (ATG), TDM (therapeutic drug monitoring), stem cell transplant (SCT), antibody, pediatrics-children

In the published article, there was an error. The unit of **AUC** used throughout the paper is incorrect.

A correction has been made to the sections “**Patients eligible for TDM and simulation for the calculated optimal dose**,” (paragraph 3) “**Therapeutic drug monitoring**” (paragraph 2) and to the footnotes of **Table 1**. The unit for **AUC** previously stated:

“AU*day/L”

The corrected unit for AUC appears below:

“AU*day/mL”

The authors apologize for this error and state that this does not change the scientific conclusions of the article in any way. The original article has been updated.

