# Supplementary material for: Therapeutic Drug Monitoring of Anti-Thymocyte Globulin in Allogeneic Stem Cell Transplantation: Proof of Concept
Source: Front Pharmacol. 2022 Mar 18;13:828094. doi: 10.3389/fphar.2022.828094 (PMC8974913; doi:10.3389/fphar.2022.828094)
Supplement: Supplementary file 1 [file Table1.DOCX]

**Therapeutic Drug Monitoring of Anti-Thymocyte Globulin in Allogeneic Stem Cell Transplantation: Proof of Concept**

J.I. Meesters-Ensing MSc, R. Admiraal, MD PhD, L. Ebskamp MSc, Amelia Lacna MSc, Jaap Jan Boelens MD PhD, C. A. Lindemans, MD PhD, Stefan Nierkens PhD

Corresponding author:

R. Admiraal, MD PhD

Princess Máxima Center for Pediatric Oncology

Heidelberglaan 25, Utrecht, The Netherlands

r.admiraal-4@prinsesmaximacentrum.nl

**Text S1 Model code for simulations**

;; 2. Description: Final model for simulations

;; x1. Author: Rick Admiraal

;----------------------------------------------------------------

; 1. use .mod extension name for control stream for NONMEM 7.2 |

; 2. use .tab as the output table extension name |

; 3. use .prn as the data input file |

;----------------------------------------------------------------

;----------------------------------------------------------------

; This is the general explaination of the model control file |

; unit of DV: U/mL |

; unit of AMT : mg |

; unit of TIME : days |

; unit of RATE : mg/day |

; unit of BW : kg |

; unit of AGE : year |

;----------------------------------------------------------------

$PROBLEM SIMULATION

;

$INPUT ID TIME DV AMT=DOSE RATE MDV EVID WT LYMNUL DGRP NRDOSE DOSIS STARTDAG

;

$DATA simulation_raw.csv IGNORE=@

;

$SUBROUTINES ADVAN6 TOL=9

;

$MODEL

NCOMP=3

COMP=(CENTRAL DEFOBS DEFDOSE)

COMP=(PERIPH)

COMP=(AUC)

;

$PK

MWT=WT/21

MLYM=LYMNUL/0.29

TVCL = THETA(1) * (MWT**THETA(9)) * (1+ MLYM*THETA(10))

CL = TVCL * EXP(ETA(1))

TVV1 = THETA(2) * (MWT**THETA(8))

V1 = TVV1 * EXP(ETA(2))

TVK21 = THETA(3)

K21 = TVK21

TVTMAX= THETA(4)

TMAX = TVTMAX

TVTM = THETA(5)

TM = TVTM * EXP(ETA(3))

TVVMAX= THETA(6)

VMAX = TVVMAX * EXP(ETA(4))

TVKM = THETA(7)

KM = TVKM * EXP(ETA(5))

K = CL/V1

S1 = V1

;-------------------------------------------------------------------------

$DES

C1 = A(1)/V1

DADT(1)= -K*A(1) -(VMAX*C1)/(KM+C1) - (TMAX*C1)/(TM+C1) + K21*A(2)

DADT(2)= (TMAX*C1)/(TM+C1) - K21*A(2)

DADT(3)= C1 ;for AUC

;-------------------------------------------------------------------------

$ERROR

IPRED = 0

IF(F.GT.0) IPRED = LOG(F) ; To prevent problems with all MDV

W=1

IRES=DV-IPRED

IWRES= IRES/W

Y = IPRED + ERR(1)

AUC = A(3)

;-------------------------------------------------------------------------

$THETA

2.15 FIX ;CL

7.83 FIX ;V1

1.19 FI ;K21

156 FIX ;Tmax

7.62 FIX ;Tm

1.79 FIX ;Vmax

1.11 FIX ;Km

1.1 FIX ;expWT op V1

0.605 FIX ;expWT op CL

0.115 FIX ;linLYM op CL

;-------------------------------------------------------------------------

$OMEGA BLOCK(2)

0.737 ;CL

0.322 0.34 FIX ;V1

$OMEGA

1.13 FIX ;Tm

0.489 FIX ;Vmax

3.12 FIX ;Km

;-------------------------------------------------------------------------

$SIGMA

0.101 FIX ;Algemene error

;-------------------------------------------------------------------------

$SIM (123456789) ONLYSIM

;

$TABLE ID TIME DV MDV IPRED WT LYMNUL DGRP NRDOSE DOSIS STARTDAG AUC

NOPRINT NOAPPEND ONEHEADER FILE=simulation.tab

**Text S2 Model code for TDM**

;; 2. Description: TDM

;; x1. Author: x

;----------------------------------------------------------------

; 1. use .mod extension name for control stream for NONMEM 7.2 |

; 2. use .tab as the output table extension name |

; 3. use .prn as the data input file |

;----------------------------------------------------------------

;----------------------------------------------------------------

; This is the general explaination of the model control file |

; unit of DV: U/mL |

; unit of AMT : mg |

; unit of TIME : days |

; unit of RATE : mg/day |

; unit of BW : kg |

; unit of AGE : year |

;----------------------------------------------------------------

$SIZES NO=800 LIM6=1000

;

$PROBLEM TDM

;

$INPUT ID TIME DV AMT RATE MDV EVID WT LYMFO NRDOSE DOSE STARTDAG

;

$DATA simulation_raw.csv IGNORE=@

;

$SUBROUTINES ADVAN6 TOL=9

;

$MODEL

NCOMP=3

COMP=(CENTRAL DEFOBS DEFDOSE)

COMP=(PERIPH)

COMP=(AUC)

;

$PK

MWT=WT/21

MLYM=LYMFO/0.290

TVCL = THETA(1) * (MWT**THETA(9)) * (1+MLYM*THETA(10))

CL = TVCL * EXP(ETA(1))

TVV1 = THETA(2) * (MWT**THETA(8))

V1 = TVV1 * EXP(ETA(2))

TVK21 = THETA(3)

K21 = TVK21

TVTMAX= THETA(4)

TMAX = TVTMAX

TVTM = THETA(5)

TM = TVTM * EXP(ETA(3))

TVVMAX= THETA(6)

VMAX = TVVMAX * EXP(ETA(4))

TVKM = THETA(7)

KM = TVKM * EXP(ETA(5))

K = CL/V1

S1 = V1

;-------------------------------------------------------------------------

$DES

C1 = A(1)/V1

DADT(1)= -K*A(1) -(VMAX*C1)/(KM+C1) - (TMAX*C1)/(TM+C1) + K21*A(2)

DADT(2)= (TMAX*C1)/(TM+C1) - K21*A(2)

DADT(3)=C1 ; AUC

;-------------------------------------------------------------------------

$ERROR

IPRED = 0

IF(F.GT.0) IPRED = LOG(F) ; To prevent problems with all MDV

W=1

IRES=DV-IPRED

IWRES= IRES/W

Y = IPRED + ERR(1)

AUC=A(3)

;-------------------------------------------------------------------------

$THETA

2.15 FIX ;CL

7.83 FIX ;V1

1.19 FIX ;K21

156 FIX ;Tmax

7.62 FIX ;Tm

1.79 FIX ;Vmax

1.11 FIX ;Km

1.1 FIX ;expWT op V1

0.605 FIX ;expWT op CL

0.115 FIX ;linLYM op CL

;-------------------------------------------------------------------------

$OMEGA BLOCK(2)

0.737 ;CL

0.32 0.339 ;V1

$OMEGA

1.13 ;Tm

0.489 ;Vmax

3.12 ;Km

;-------------------------------------------------------------------------

$SIGMA

0.101

;-------------------------------------------------------------------------

$EST MAXEVAL=0 METHOD=1 POSTHOC INTERACTION NSIG=3 SIGL=9

;

$TABLE ID TIME WT AUC LYMFO CL V1 K21 TMAX VMAX KM TM

IPRED CWRES MDV NRDOSE DOSE STARTDAG

NOPRINT ONEHEADER FILE=output.tab
